# Pathogenic Role of Human Rhinovirus Mono-Infection in Pediatric Lower Respiratory Tract Infection

**DOI:** 10.7759/cureus.60032

**Published:** 2024-05-10

**Authors:** Monalisa Mohanty, Baijayantimala Mishra, Bhagirathi Dwibedi, Rashmi R Das, Sailendra Panda, Debashis Santra, Madhab Charan Mandal, Prabhudutta Mamidi, Krishna M Gulla

**Affiliations:** 1 Microbiology, All India Institute of Medical Sciences, Bhubaneswar, Bhubaneswar, IND; 2 Pediatric Medicine, All India Institute of Medical Sciences, Bhubaneswar, Bhubaneswar, IND; 3 Pediatrics, All India Institute of Medical Sciences, Bhubaneswar, Bhubaneswar, IND; 4 Virology, All India Institute of Medical Sciences, Bhubaneswar, Bhubaneswar, IND

**Keywords:** human rhinovirus, cycle threshold (ct) value, lower respiratory tract infection, pediatric, pathogen, mono-infection

## Abstract

Background and objective

Human rhinovirus (HRV) is one of the leading causes of pediatric respiratory tract infection with a prevalence rate of 30-50%, mostly affecting children below five years of age and causing a substantial amount of economic loss. In children, it can alone or as a co-infection, cause a wide range of symptoms from mild to life-threatening ones. With the above background, the current study was carried out to emphasize the role of HRV mono-infection in pediatric acute respiratory tract infections by correlating clinical and molecular laboratory findings.

Methods

This study was carried out in a tertiary care teaching hospital over a duration of four years (March 2019-October 2023). Children up to 14 years of age visiting the outpatient department or admitted to the ward with diagnoses of acute respiratory tract infections (ARTIs) were included. The clinical and laboratory data were retrieved and analyzed. A nasopharyngeal swab (NPS) or throat swab (TS) was collected and sent to the Microbiology laboratory maintaining the cold chain. Nucleic acid was extracted and subjected to multiplex real-time polymerase chain reaction (RT-PCR).

Result

Of the 245 samples tested for the respiratory viral pathogen, 52 samples tested positive for HRV, of which 27 had HRV mono-infection. The clinico-demographic details of these 27 patients were studied in detail. The majority of the cases (24/27; 88.8%) were less than five years of age. Fever and shortness of breath were the most consistent symptoms in all. Nineteen (19/27; 62.9%) HRV mono-infection cases had underlying co-morbidities, all requiring respiratory support. The HRV mono-infection cases either developed bronchiolitis, lower respiratory tract infection, or pneumonia. All mono-infection cases had cycle threshold value (Ct) < 25, while the Ct value of HRV was > 30 in co-infection with other viruses.

Conclusion

Mono-infection of HRV in under-five children with underlying comorbidities and a lesser Ct value indicates severe disease manifestation and should be dealt with more cautiously.

## Introduction

Human rhinovirus (HRV) is one of the common causes of the common cold in adults and children [1-3]. More than half of the cold-like complaints are attributed to HRV and as a result, it costs a substantial amount of economic loss annually on frequent medical visits and missed days at work and school [4]. Although most of the episodes of illness caused by HRV are mild infections in the upper airways, they can cause infections in the lower respiratory tract. It is responsible for exacerbations of asthmatic attacks and chronic obstructive pulmonary disease (COPD) in 10-40% of cases infected with HRV [1-4]. To date, specific antiviral therapies and vaccines against HRV continue to be evasive, which again adds to the magnitude of the problem [2].

Rhinovirus belongs to the genus Enterovirus of the Picornaviridae family. Advancements in molecular diagnostic approach have upgraded our knowledge regarding the genomic structure of HRV and have led to the characterization of HRV into more than 160 serogroups and three genotypes - A, B, and C [3,5]. Though HRV is known to be associated with upper respiratory tract infection (URTI), genotype C is documented to be associated with acute exacerbation of wheezing episodes in asthma and lower respiratory tract infection (LRTI) particularly in small children [6]. Even though in immunocompetent individuals, viral shedding usually lasts for 10 to 14 days, it is not always associated with respiratory symptoms [3,7,8]. However, prolonged rhinoviral shedding over a period of 5-6 weeks has been observed in young children after a symptomatic episode, and chronic rhinovirus carriage with a viral shedding duration over 4-12 months has been reported in immunocompromised patients [3].

HRV has been isolated as a co-pathogen with other viral and bacterial agents [3,9], which again creates a controversy on its exclusive pathogenic role of clinical significance. However, the association of HRV mono-infection with LRTI and severe episodes of asthma warrants further investigations on the pathogenic role of this virus [6]. With this background, the present study was conducted to emphasize the role of HRV mono-infection in pediatric acute respiratory tract infections (ARTIs) correlating clinical and molecular laboratory investigations.

## Materials and methods

Study design

This was a retrospective study and it was carried out in the Department of Microbiology and Pediatrics of a tertiary care teaching hospital in Eastern India.

Study population

Children up to 14 years of age visiting the outpatient department of pediatrics or admitted to the ward with a diagnosis of acute respiratory tract infections (ARTIs), i.e., either having upper respiratory tract infection (URTI) or lower respiratory tract infection (LRTI) were included in the study. Institutional ethical approval was taken before starting the study with the approval number T/IM-NF/Micro/23/102. The clinical and laboratory data over a duration of four years (March 2019-October 2023) were retrieved and analyzed.

Microbiological testing methods

Clinical Specimen

For microbiological diagnosis in the case of ARTIs, samples like throat swab (TS), nasopharyngeal swab (NPS), broncho-alveolar lavage (BAL), or sputum are needed. In the present study, throat swabs (TS) or nasopharyngeal swabs (NPS) were collected from children with suspected viral ARTI as per the WHO criteria [10]. The samples were then sent to the Microbiology laboratory in the viral transport medium (VTM) maintaining the cold chain. Nucleic acid was extracted and the rest of the samples were aliquoted and stored at -80˚C for any further testing. Simultaneously, for routine bacteriological investigations, blood samples were collected in the blood culture bottles and sent for culture and sensitivity testing.

Nucleic Acid Amplification Test

Extracted nucleic acid was subjected to multiplex real-time polymerase chain reaction (RT-PCR) using Fast Track Diagnostics (FTD, Fast-Track Diagnostics, Luxemburg) Respiratory Pathogens 21 kit with the detection capability of 20 viral and one bacterial pathogen such as including influenza A (IAV), influenza B (IBV), influenza A [IAV(H1N1)sw1], parainfluenza virus (HPIV-1, HPIV-2, HPIV-3, HPIV-4), human coronaviruses (HCoV 229E, HCoV NL63, HCoV OC43, HCoV HKU1), human rhinovirus (HRV), human bocavirus (HBoV), human respiratory syncytial virus (HRSV A & B), adenovirus (HAdV A & B), human metapneumovirus (HMPV A & B), human parechovirus (HPeV), Epstein-Barr virus (EV), and Mycoplasma pneumoniae, respectively. Real-time PCR was done either on Bio-Rad CFX 96 or Quant Studio 5 Dx (Thermo Fisher Scientific®) according to the manufacturer’s instructions. The criteria for a valid run were, (i) for the positive control (PC): Amplification present (Ct value <33), (ii) for negative control (NC): No amplification except IC (Ct value <33), (iii) Positive result: Amplification present and IC (Ct<33).

Clinical Workup

Radiological investigations such as Chest X-ray were done in children with LRTI. The clinical and laboratory details were retrieved and analyzed. The case details of the patients who were detected to have HRV infection were studied in detail. The data included detailed descriptions of demography, clinical presentation, investigations, and outcomes.

## Results

**Figure 1:**
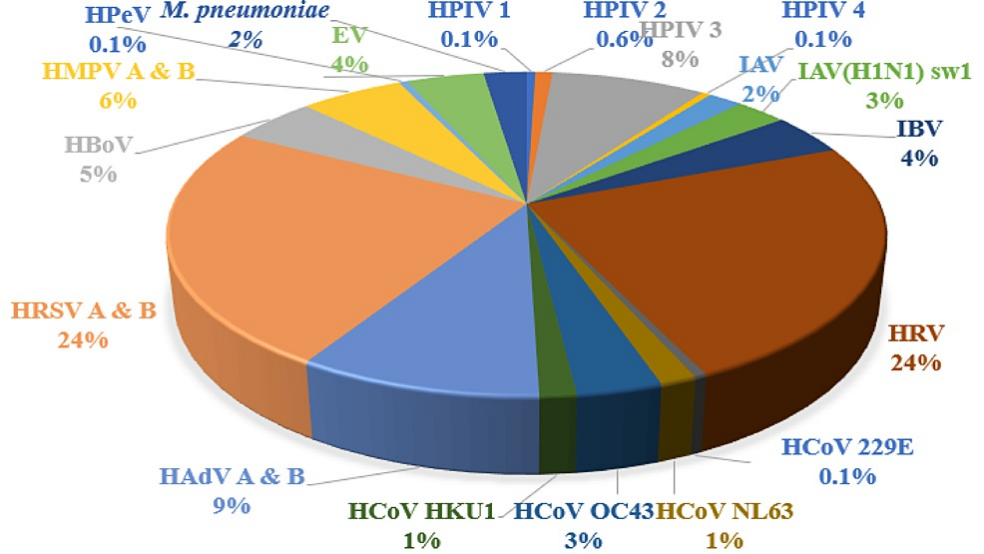
Details of the positive respiratory pathogens isolated Influenza A (IAV), influenza B (IBV), influenza A [IAV(H1N1)sw1], parainfluenza virus (HPIV-1, HPIV-2, HPIV-3, HPIV-4), human coronaviruses (HCoV), human rhinovirus (HRV), human bocavirus (HBoV), human respiratory syncytial virus (HRSV A & B), adenovirus (HAdV A & B), human metapneumovirus (HMPV A & B), human parechovirus (HPeV), Epstein-Barr virus (EV)

Of the 245 samples tested for respiratory viral pathogen, 216 tested positive for different viral pathogens, of which 52 samples tested positive for HRV (Figure 1).

Out of the 52 HRV-positive cases, 27 had HRV mono-infection, and the rest of the cases had co-infections with other viruses. The clinico-demographic details of the 27 patients with HRV mono-infection are described in Table 1.

**Table 1:** Clinico-epidemiological details of 27 HRV mono-infection cases SOB – Shortness of breath, CDH – Congenital diaphragmatic hernia, CLD – Chronic lung disease, CXR – Chest X-ray, RT PCR – Real-time polymerase chain reaction, LRTI – Lower respiratory tract infection, VSD – Ventricular septal defect, PDA – Patent ductus arteriosus, BPD – Broncho-pulmonary dysplasia, CCF – Congestive cardiac failure, y – year, mo – month, wk – week, d – day.

Serial Number	Age	Sex	Symptoms	Co-morbidity	Blood culture	CXR	Diagnosis	Clinical Management	Outcome
1	3 mo	M	Fever (5 days), SOB	-	Sterile	Normal	LRTI	Administration of antibiotics, oxygen & Salbutamol	Discharged
2	1.6 y	M	Fever (5 days), SOB	Recurrent wheeze	Sterile	Normal	LRTI	Administration of oxygen & Salbutamol	Discharged
3	10 y	F	Fever, SOB	Mild asthma	Sterile	Patchy infiltrate in both lungs	LRTI	Administration of antibiotics, oxygen, steroids & Salbutamol	Discharged
4	1 mo	M	Fever, SOB	-	Sterile	Peribronchial cuffing	Viral Pneumonia	Administration of antibiotics, oxygen & Salbutamol	Discharged
5	11 mo	M	Fever (7 days), SOB, cough, rhinorrhea	- cyanotic heart disease (VSD)	Sterile	Patchy infiltrate in both lungs	LRTI	Administration of antibiotics & oxygen	Discharged
6	1 y	M	Fever (4 days), cough, SOB	-	Sterile	Normal	LRTI	Administration of antibiotics & oxygen	Discharged
7	1 y	F	Fever (7 days), SOB		Sterile	Normal	LRTI	Administration of antibiotics & oxygen	Discharged
8	10 y	F	Fever (3 days), SOB		S. aureus (MSSA)	Patchy infiltrate in both lungs	LRTI	Administration of antibiotics & oxygen	Discharged
9	9 y	M	Fever (2 days), SOB	Infrequent relapsing nephrotic syndrome	Sterile	Normal	LRTI	Administration of antibiotics, oxygen & steroids (stress dose)	Discharged
10	1 y	M	Fever (7 days), SOB	-	Sterile	Normal	LRTI	Administration of antibiotics, oxygen & Salbutamol	Discharged
11	4 y	M	Fever (3 days), SOB	CLD	Sterile	Features of ARDS	LRTI+ CLD	Administration of antibiotics & oxygen. Required mechanical ventilation	Discharged
12	2 y	M	Fever (3 days), SOB	Bronchiectasis (non-cystic fibrosis)	S. aureus (MSSA)	Patchy opacities in both lungs	LRTI + Bronchiectasis	Administration of antibiotics & oxygen, & Salbutamol	Discharged
13	1 y	M	Fever (6 days), SOB		Sterile	Normal	LRTI		Discharged
14	1 y	M	Fever (4 days), SOB		Sterile	Hyperinflation of both lungs	LRTI	Administration of antibiotics & oxygen, & Salbutamol	Discharged
15	7 mo	M	Fever (5 days), SOB	Preterm with BPD	Sterile	Hyperinflation of both lungs with patchy infiltrates	LRTI	Administration of antibiotics & oxygen, & Salbutamol	Discharged
16	2.5 mo	F	Fever (3 days), SOB	Neonatal cholestasis syndrome	Sterile	Hyperinflation of both lungs with patchy infiltrates	Viral pneumonia	Administration of antibiotics & oxygen	Discharged
17	1 y	M	Fever (4 days), SOB	Recurrent wheeze	Sterile	Patchy interstitial infiltrates	LRTI	Administration of antibiotics & oxygen	Discharged
18	3 mo	F	Fever (4 days), SOB	Obstructive uropathy	Sterile	Patchy infiltrates in both lungs	Bronchiolitis	Administration of antibiotics & oxygen, & Salbutamol	Discharged
19	2 wk	F	Fever (3 days), SOB	Preterm baby with growth restriction	Sterile	Patchy consolidation	Pneumonia	Administration of antibiotics & oxygen, & Salbutamol	Discharged
20	6 mo	M	Fever (4 days), SOB		Sterile	Normal	LRTI	Administration of antibiotics & oxygen, Salbutamol	Discharged
21	2 mo	F	Fever (5 days), SOB	Preterm with PDA	Sterile	Perihilar cuffing	LRTI	Administration of antibiotics, oxygen & decongestive measures for CCF	Discharged
22	5 mo	M	Fever (3 days), SOB		Sterile	Normal	Bronchiolitis	Administration of oxygen & Salbutamol	Discharged
23	1 y	M	Fever (4 days), SOB	Birth asphyxia with developmental delay	Sterile	Patchy infiltrates in both lungs	LRTI	Administration of antibiotics, oxygen, & Salbutamol	Discharged
24	1 mo	M	Fever (4 days), SOB	Preterm with BPD	Sterile	Patchy infiltrates	Bronchiolitis	Administration of oxygen & Salbutamol	Discharged
25	1 mo	M	Fever (3 days), SOB	-	Sterile	Patchy infiltrates	Bronchiolitis	Administration of oxygen & Salbutamol	Discharged
26	8 mo	M	Fever (4 days), SOB	Birth asphyxia with developmental delay	Sterile	Hyperinflation of both lungs with interstitial infiltrates	LRTI	Administration of antibiotics, oxygen, Salbutamol	Discharged
27	25 d	M	Fever (5 days), SOB, Cough	Neonatal cholestasis syndrome	Sterile	Patchy infiltrates in both lungs	Viral Pneumonia	Administration of antibiotics, oxygen	Discharged

The majority of the cases (24/27; 88.8%) belonged to the age group of less than 5 years, of which, 14 cases (51.8%) were of less than 1 year of age. Fever (an axillary temperature of ≥100.40F) and shortness of breath (SOB) were the most consistent symptoms in all HRV-positive cases. Male (20/27) to female (7/27) ratio was 2.8.

**Figure 2:**
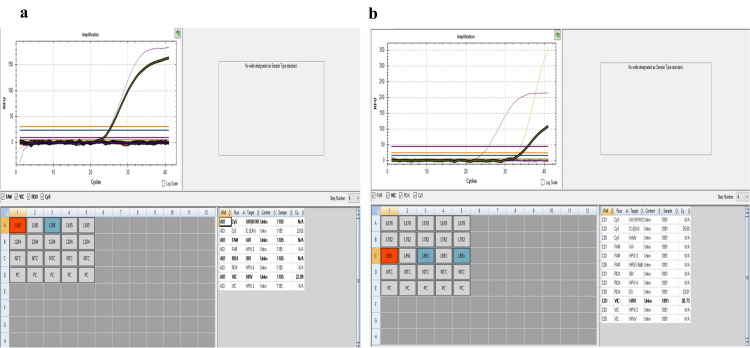
(a) HRV mono-infection. (b) HRV + EV co-infection HRV - Human rhinovirus, EV - Epstein-Barr virus

In HRV mono-infection cases (n=27), 19 (62.9%) cases had underlying co-morbidities, either having underlying heart disease, lung pathology, preterm, liver, or kidney issues, with all requiring respiratory support. Two of the HRV mono-infection cases developed pneumonia. All the HRV mono-infection cases had cycle threshold value (Ct) < 25, while the Ct value of HRV was > 30 in co-infection with other viruses (Figure 2).

The co-infected cases, particularly with other Epstein-Barr virus with Ct value > 30 had URTI manifesting in the form of running nose, and nasal congestion, while the HRV mono-infection cases had severe clinical signs and symptoms and landed in LRTI requiring respiratory assistance and other supportive care.

The cases were detected throughout the year with maximum (23/27: 85.1%) cases during the winter season. They were managed with antihistamines, oxygen administration, steroids, and salbutamol. The final diagnoses of these cases were made as bronchiolitis, lower respiratory tract infection (LRTI), or pneumonia due to viral etiology. No death was reported among these mono-infection cases and all of them were discharged.

## Discussion

The present hospital-based study supported that HRV is the most common viral agent detected in pediatric acute LRTI cases with a prevalence of 24.1% (52/216 positive cases). Alsayed et al. and Aizawa et al., in their studies, also reported HRV as the most common viral agent with different prevalence rates, 16.5% and 85.2%, respectively [11,12]. The difference in prevalence rates might be due to the difference in the size of the study, population, geographical location, diagnostic methods, and the period of study. The HRV mono-infection cases in the present study were 51.9% and of the mono-infection cases, 88.8% were less than five years of age, and more than 50% were infants (<1 year age). In the earlier studies on HRV, the majority of the children were less than 3 years of age. This study supports the results of the previous studies, it is observed that HRV is one of the common viruses responsible for childhood LRTIs.

Calvo et al. in their studies, between September 2003 and July 2005, showed that shortness of breath, wheezing, and exacerbation of asthma episodes were due to various viral pathogens with influenza virus, human parainfluenza virus (HPIV), human metapneumovirus (HMPV), and respiratory syncytial virus (RSV) being the major causes of hospitalization in children. But later, a study by Aizawa et al. between January 2020 and September 2022, reported that HRV was one of the common agents responsible for childhood ARTIs [12,13]. In HRV mono-infection cases in the present study, it was seen that all the cases experienced shortness of breath and required respiratory support. The mechanism behind these symptoms can be explained by the fact that HRV infection triggers airway inflammation by increasing the histamine and tumor necrosis factor α (TNF-α) levels in the bronchoalveolar lavage fluid and enhancing eosinophil recruitment into the airways [14]. Several other investigators have found out that HRV infection differentially increases or decreases the levels of different inflammatory mediators in URTIs and LRTIs; the levels of kinins or cytokines (interleukin-1 [IL-1], IL-8, and IL-11) increase and lymphocyte level decreases in HRV infection [15]. Fraenkel et al. in their study noticed that there was a significant increase in the levels of submucosal CD3+ lymphocytes and epithelial eosinophils in bronchial mucosal biopsy specimens with experimental HRV infection. These increased inflammation levels can be correlated with hyperresponsiveness to histamines [16]. Furthermore, the lower Ct value (<25) in all the HRV mono-infection cases in the present study indirectly supports the evidence of suspecting more severe clinical manifestations of HRV-infected patients. Previous studies by Esneau et al. and Wishaupt et al. have also reported that a lower Ct value corresponds to a higher viral load particularly in HRV genotype C with a higher symptom score [17,18].

HRV replicates efficiently at lower temperatures (preferably at 33-35˚c) and therefore previously it was thought to cause only URTIs, but recently the molecular diagnostic modalities have proven its association with LRTIs at core body temperature of 37˚c and wheezing episodes in children, particularly with those having underlying co-morbid conditions like asthma, cystic fibrosis, and bronchopulmonary dysplasia [17]. In the present study, too, more than 70% of cases had an underlying co-morbid condition. The transmission of HRV infections is perennial with a slight increase in transmission rate being observed in autumn and spring, but maximum cases have been reported during the winter and spring seasons [19-22]. In the present study also >85% of cases were detected during the winter season. The peak incidences of HRV cases depend on the seasonal and geographical distribution of circulating strain, and the severity of illness. Substantial evidence shows the surge in HRV infection at the re-opening of the educational institution after the holidays, which is mostly due to an interim absence of exposure during holidays followed by a fleeting period of susceptibility to HRV infections, particularly in small children [21,22].

HRV genotyping studies can be implicated in investigating the genetic diversity of HRV strains in respiratory samples sent for diagnostic testing and their relationship with the severity of different respiratory diseases [12]. Recent studies on the genotype of HRV had reported a third genotype along with genotypes A and B, i.e., HRV genotype C (HRV-C). HRV-C has been associated with various clinical outcomes ranging from mild or even asymptomatic infections to acute lower respiratory tract illnesses including bronchiolitis, exacerbation of asthma, recurrent wheezing, and pneumonia [12,21,23]. Additionally, genotyping studies as done by Pierangeli et al. and Bochkov and Gern can help the researchers in understanding the virulence mechanisms, and the development of specific vaccines and antiviral agents [22,23]. The present study, however, could not perform the HRV genotyping study, which is a limitation of this study.

## Conclusions

HRV is one of the major viral pathogenic agents causing LRTIs in pediatric groups. The study supports additional caution when treating the mono-infection of HRV in under-five children with underlying comorbidities and lesser Ct value where it indicates severe disease manifestation. Genotype studies need to be conducted for further understanding of the correlation of different genotypes with different clinical manifestations, development of vaccines, and specific antiviral agents.
